# Neurological paraneoplastic syndromes in patients with breast and gynecological cancer—a case series and structured literature review

**DOI:** 10.1007/s00404-026-08513-0

**Published:** 2026-08-07

**Authors:** Damaris Erhardt, Maria Karakitsiou, Claudia Rauh, Julian Wampfler, Uzeyir Ahmadli, Franziska Siegenthaler, Michael D. Mueller, Sara Imboden, Flurina A. M. Saner

**Affiliations:** 1 Department of Gynecology and Obstetrics, Bern University Hospital and University of Bern, Bern, Switzerland; 2 Department of Oncology, Bern University Hospital and University of Bern, Bern, Switzerland; 3 Department of Neuroradiology, Bern University Hospital and University of Bern, Bern, Switzerland

**Keywords:** Breast cancer, Anti-Yo, Anti-amphiphysin, Autoimmune cerebellar degeneration, Autoimmune encephalitis

## Abstract

**Purpose:**

Neurological paraneoplastic syndromes (PNS) occur in less than 1% of patients with cancer but carry a significant clinical burden. Prognosis strongly depends on timely diagnosis and treatment. This study presents an illustrative case series and a structured review of neurological PNS in a gynecological context. This case series describes four patients (ages 61–79) with neurological PNS with underlying breast or pelvic malignancies, diagnosed with anti-amphiphysin encephalitis, autoimmune myeloradiculitis, and paraneoplastic cerebellar ataxia.

**Methods:**

A structured literature review focusing on neurological PNS in gynecological and breast oncology with at least two participants was conducted using PubMed and MEDLINE. The results are synthesized, focusing on diagnostic workup and clinical findings.

**Results:**

The review identified 5 original studies comprising 72 patients. Most cases were associated with breast cancer; however, largely attributable to one large study. Neurological PNS should be considered once alternative etiologies have been excluded through appropriate diagnostic testing. Most often this includes neurologic imaging. Diagnosis requires antibody testing and subsequent malignancy investigation, guided by the specific antibody profiles. Main limitations were the influence of one large study with the remaining sample sizes being small, as well as the retrospective study designs.

**Conclusion:**

Due to their rarity and complexity, neurological PNS pose significant diagnostic and treatment challenges. Since neurological symptoms often precede the cancer diagnosis, thorough diagnostics and prompt treatment are essential, as prognosis is time-sensitive. Multidisciplinary care is crucial, and further research is necessary to improve understanding and management of these syndromes.

## What does this study add to the clinical work

**Table:** 

Detecting rare neurological paraneoplastic syndromes in gynecological cancers remains challenging due to their diverse symptom profiles. By combining a structured review with real-world cases, this study enhances clinical awareness and highlights the critical importance of early diagnosis and prompt interdisciplinary management.	

## Introduction

Paraneoplastic syndromes (PNS) are rare conditions that occur in up to 8% of patients with cancer [1]. First described in the 1940 s, certain tumors induce clinical symptoms unrelated to the tumor itself, its metastases or its treatment. These symptoms derive from an immune-modulated response and a cross-reaction with the body’s own antigens, or by cytokines and hormones secreted by the tumor itself. Cross-reactions against neurologic antigens cause neurological PNS and occur in less than 1% of patients with cancer [2]. Breast and gynecologic cancers are the second most frequent underlying neoplasms after lung cancer [3]. These syndromes sometimes precede the diagnosis of the neoplasm or signal its recurrence, hence gynecologists play a critical role in early detection. Suspicion should be triggered, when new neurologic symptoms cannot be otherwise explained [4]. Therefore, primarily, alternative causes need to be excluded, which often entail neurologic imaging such as brain MRI, for example for direct cancer affection [5]. A high-risk neurologic phenotype should immediately trigger a search for an underlying malignancy. Antibodies serve as additional information to guide the diagnostic investigation toward specific organ systems [6]. Their management primarily focuses on treating the underlying malignancy and secondarily includes immunosuppressive or modulatory treatment. PNS often impacts a patient’s clinical course profoundly, affecting treatment outcome, prognosis, and sometimes mimicking metastasis.

This case series and the following structured literature review aim to raise awareness and inform clinical practice by presenting typical clinical presentation and findings, as well as the diagnostic process, in women with breast and gynecological cancers affected by neurological PNS. To our knowledge, no literature review combined with a case series, focusing exclusively on the neurological manifestations of paraneoplastic syndromes within this specific oncological context has been published to date.

## Illustrative case series

In this consecutive case series, patients treated for a breast or gynecological cancer at the University Hospital Bern between 2020 and 2024 and diagnosis of a neurological PNS were included. Corresponding clinical and radiologic findings of a PNS, which could not be explained by other neurological conditions, and presence of serum or cerebrospinal fluid antibodies were additional inclusion criteria. Data were extracted from our electronic medical database in patients with written general consent. According to the local ethics committee, approval for publication of this case series was not necessary.

Overall, four patients met inclusion criteria for our case series (Table 1).

**Table 1:** Patient characteristics and clinical course in a case series of four patients with paraneoplastic syndromes due to a gynecological or breast cancer

Case	Age	Cancer type	FIGO^a^ stage	Cancer biology	PNS^b^ type	Antibody findings (specimen)	Radiologic findings	Clinical presentation	Delay between PNS^b^ and cancer diagnosis	PNS^b^ treatment	PNS^b^ course	PFS^c^ and OS^d^
1	61	Breast cancer	IIIA	NST^e^, ER^f^-positive, PR^g^-positive, Ki-67 20%, HER2 negative, G3	Encephalitis	Anti-amphiphysin (CSF^h^ + S^i^)	MRI^j^ head: cerebellar atrophy	Rapidly progressing vertigo, gaze-evoked nystagmus, unsteady gait and tendency to fall, ataxia, and hypesthesia	7 days	5 days of methylprednisolone. IVIG^k^ discontinued after 18 months	PNS^b^ treatment response: no improvement with methylprednisolone. Cancer therapy response: no change in balance issues, numbness in the left arm, and ataxia. Complication: visual impairment related to IVIG^k^ administration. Long-term follow-up: significant functional improvement 24 months after IVIG^k^ discontinuation, regaining ability to walk short distances; no oncological relapse	Ongoing
2	79	Ovarian cancer	IVB	High-grade serous, p53 mutated	Cerebellar ataxia	anti-Yo (CSF^h^ + S^i^)	MRI^j^ head: cerebellar atrophy	Truncal ataxia, gait disturbance, and sensorimotor polyneuropathy	17 months	8 days of methylprednisolone. 4 days of IVIG^k^. Corticosteroid for 15 months. Mycophenolat mofetil ongoing	PNS^b^ treatment response: improved head tremor; worsening stance and gait ataxia; gaze-evoked nystagmus; progressive cerebellar degeneration observed on MRI^j^. Cancer therapy response: no change in severe ataxia, scanning speech, oculomotor dysfunction, or axonal sensorimotor polyneuropathy	Ongoing
3	62	Endometrial cancer	IIIC1	High-grade serous, p53 mutated	Meningoencephalitis with cerebellar involvement	anti-Yo (CSF^h^ + S^i^)	MRI^j^ head: cerebellitis, 5 months later MRI^j^ head: cerebellar degeneration	Headaches, ataxia affecting the right extremities, vertigo, down-beat nystagmus, and square-wave jerks	16 days	5 days of methylprednisolone. 4 cycles of 5 days IVIG^k^ over 6 months. Rituximab ongoing	Overall: progression, particularly with worsening dysphagia and development of tetraparesis	23 months PFS^c^, 24 months OS^d^
4	63	Breast cancer	IIB	NST^e^, ER^f^-positive, PR^g^-positive, Ki-67 50%, HER2 negative, G3	Myeloradiculitis	Anti-GM2 (S^i^)	MRI^j^ head: normal. MRI^j^ spinal axis: contrast enhancement of the cauda equina, indicative for inflammatory cause	Facial nerve palsy, positive pyramidal tract signs, and atrophic tetraparesis	No delay	2 cycles of 3 days IVIG^k^. pause and restart of therapy after 9 months	Overall: stable sensory-motor symptoms in the lower extremities, with slightly increased unilateral hand weakness	12 months PFS^c^, 27 months OS^d^

*FIGO*^a^ International Federation of Gynaecology and Obstetrics, *PNS*^b^ paraneoplastic syndrome, *PFS*^c^ progression free survival, *OS*^d^ overall survival, *NST*^e^ no special type, *ER*^f^ estrogen receptor, *PR*^g^ progesterone receptor, *CSF*^h^ cerebrospinal fluid, *S*^i^ serum, *MRI*^j^ magnetic resonance imaging, *IVIG*^k^ intravenous immunoglobulin

### Case 1: anti-amphiphysin encephalitis

A 61-year-old patient was referred to our neurology department due to rapidly progressing vertigo and unsteady gait. Clinically, we observed a paracerebellar syndrome, characterized by gaze-evoked nystagmus with a tendency to fall, ataxia, and hypesthesia. A head MRI revealed cerebellar atrophy without any signs of cerebellitis. A computed tomography (CT) led to a suspicion of unilateral breast cancer with metastasis in the axillary lymph nodes, the adrenal glands, and the spleen. Subsequent mammography, ultrasonography, and biopsy confirmed the diagnosis of a poorly differentiated invasive breast cancer. The paracerebellar syndrome was confirmed to be paraneoplastic in origin, supported by the presence of anti-amphiphysin antibodies in both cerebrospinal fluid and serum. A multidisciplinary tumor board recommended neoadjuvant chemotherapy. However, after receiving three doses of paclitaxel, the patient developed an epidural abscess with paravertebral muscular and periosteal involvement, requiring surgical drainage and antibiotic therapy. This prompted an earlier surgical cancer treatment by simple mastectomy with axillary dissection, followed by adjuvant antihormone therapy with letrozole for her stage ypT2 ypN1a cancer. The subsequent therapy with a cyclin-dependent kinase 4/6 (CDK4/6) inhibitor was poorly tolerated and discontinued. Initially, the patient was treated with a 5-day course of methylprednisolone for the PNS encephalitis, followed by intravenous immunoglobulin (IVIG) perfusions every 6 weeks. Initially, the PNS symptoms showed moderate improvement with IVIG therapy but later stabilized, with persistent balance issues, numbness in the left arm, and moderate ataxia. At this stage, she remained dependent on a wheelchair when leaving the house. After approximately 18 months, IVIG therapy was discontinued due to visual disturbances following an infusion and because the progression of PNS symptoms had plateaued. Notably, 2 years after the cessation of IVIG therapy, PNS symptoms have improved with the patient regaining the ability to walk up to 100 m. To date, there is no evidence of cancer relapse.

### Case 2 and 3: anti-Yo-associated cerebellar syndromes

**Fig. 1:**
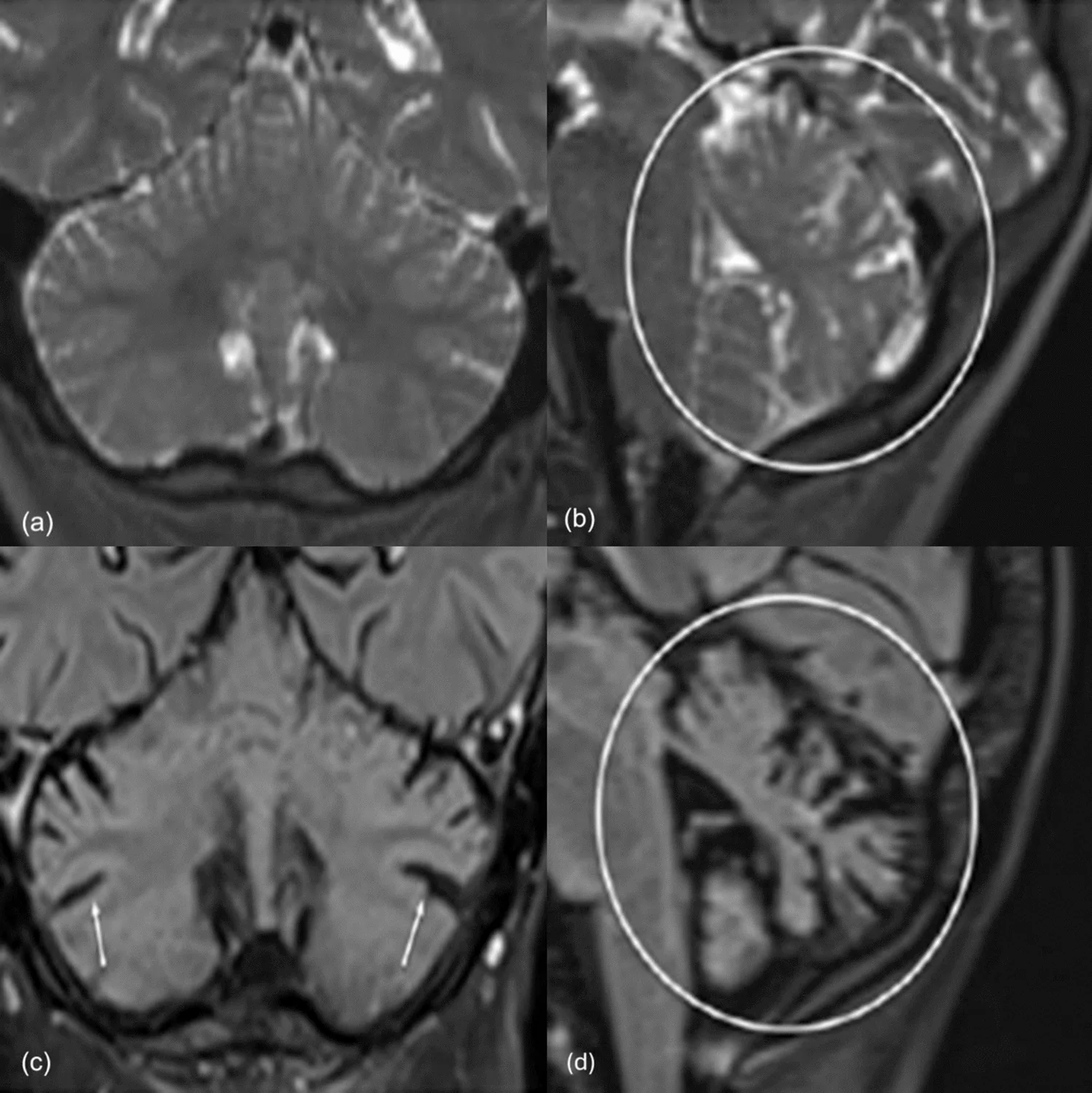
Head MRI of Case 2 including (**a**–**b)** T2-weighted images one year before diagnosis for comparison and coronal post-contrast fluid-attenuated inversion recovery (FLAIR) (**c**), sagittal 3D FLAIR (**d**), showing cerebellar atrophy indicated by the white circle in d compared to b

A 79-year-old patient diagnosed with autoimmune cerebellar ataxia presented with truncal ataxia, gait disturbance, and sensorimotor polyneuropathy. Anti-Yo antibodies were found in her serum and cerebrospinal fluid and a head MRI revealed progressive cerebellar atrophy (Fig. 1). Initial malignancy screening, including a CT scan of the thorax and abdomen, mammography, and positron emission tomography (PET)-CT, was negative. 6 months later, the patient developed ascites; however, cytological analysis revealed no malignant cells. The ascites was attributed to portal hypertension secondary to chronic portal vein thrombosis following a splenectomy performed at age 30. Following multiple diagnostic paracenteses and repeated imaging over a period of 17 months, a follow-up CT scan of the abdomen and thorax identified inguinal lymphadenopathy. A biopsy of the lymph nodes showed a poorly differentiated adenocarcinoma, positive for WT1, p53, and CK7, indicative of metastasis from a high-grade serous ovarian cancer. Neoadjuvant chemotherapy with carboplatin and paclitaxel was initiated as recommended by the institutional multidisciplinary tumor board, having considered the patient’s poor general condition. The cerebellar degeneration was treated with IVIG, prednisolone, and mycophenolate mofetil. Under this therapy, mainly the head tremor improved with worsening of the gait and stance ataxia and nystagmus. However, a few months later, the patient chose to discontinue the chemotherapy, opting instead for palliative care.

**Fig. 2:**
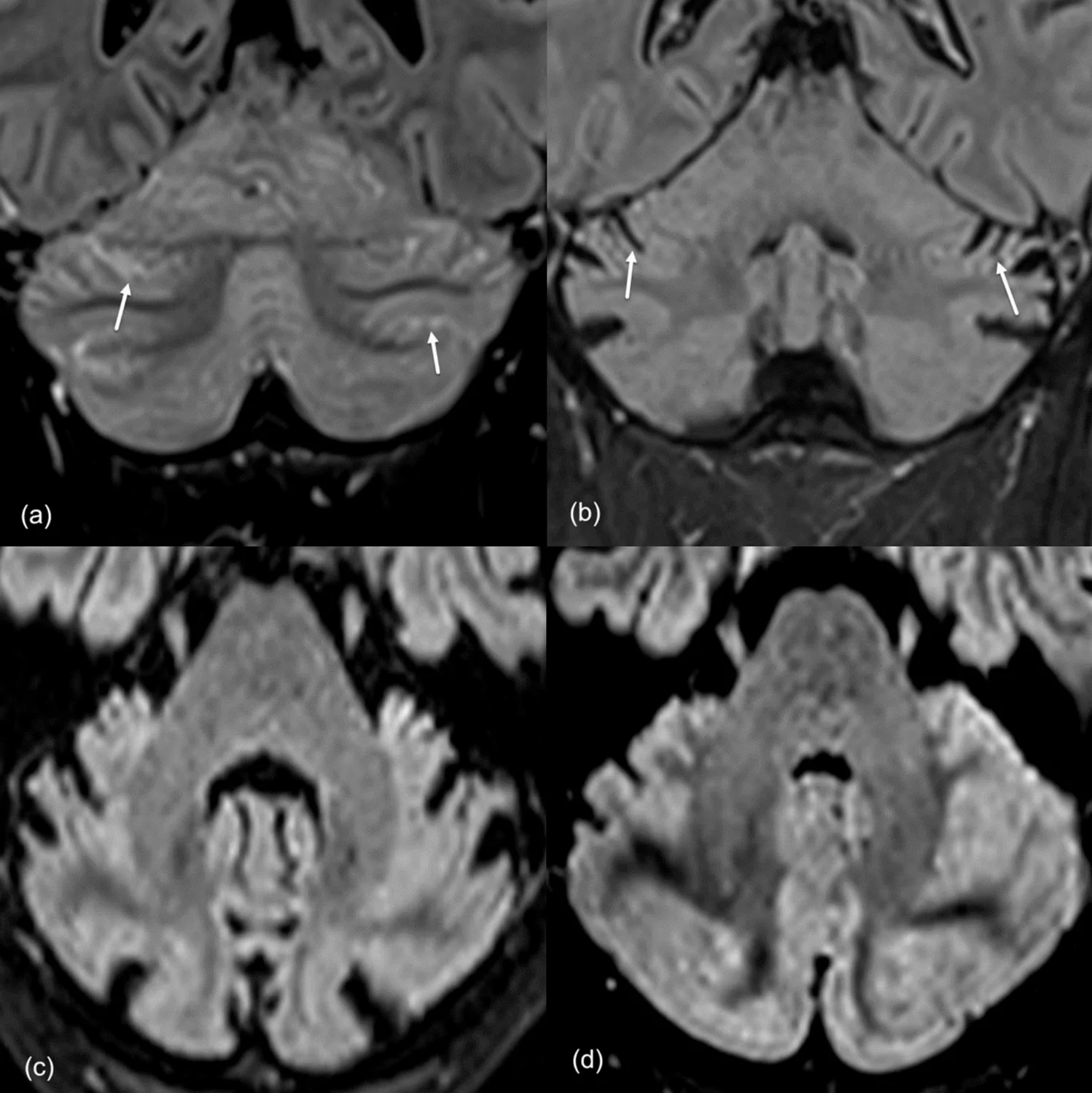
Head MRI of Case 3 showing **a** leptomeningeal enhancement indicated by white arrows in contrast-enhanced cor fatsat FLAIR, **b** 5-month follow-up showing resolution indicated by white arrows, and **c**, **d**, axial FLAIR sequence secondary cerebellar atrophy at admission and follow-up

A second patient was diagnosed with anti-Yo paraneoplastic meningoencephalitis concurrent with the diagnosis of locally advanced serous p53 mutated endometrial cancer with ovarian metastases. This 62-year-old woman underwent hysterectomy, bilateral adnexectomy, sentinel lymphadenectomy, and omentectomy. Around the same time, the patient began experiencing symptoms including headaches, ataxia affecting the right extremities, vertigo, down-beat nystagmus, and square-wave jerks. A head MRI showed a cerebellar atrophy (Fig. 2). Further examinations revealed anti-Yo antibodies in the cerebrospinal fluid as well as in the serum, confirming paraneoplastic anti-Yo meningoencephalitis with cerebellar involvement. The multidisciplinary tumor board recommended adjuvant radiochemotherapy with cisplatin, brachytherapy, and subsequent adjuvant chemotherapy with carboplatin and cisplatin for FIGO IIIC1 disease. The paraneoplastic syndrome was treated with prednisolone, IVIG, and Rituximab. During the course of the disease, a vesicoperitoneal fistula developed, necessitating the placement of a bladder catheter and prophylactic treatment with fosfomycin. The patient’s condition progressed to a challenging palliative situation, requiring the coordinated care of gynecologists, urologists, oncologists, and neurologists. Therefore, the radiation therapy was not performed. Approximately 17 months after completion of chemotherapy, the tumor recurred in the paramedian pelvic region and in the pelvic lymph nodes. Along with the progression of the PNS symptoms, including aggravated dysphagia and the development of tetraparesis, the patient’s condition deteriorated, and she passed away 2 years after initial cancer diagnosis.

### Case 4: autoimmune myeloradiculitis

A 63-year-old patient was diagnosed with a paraneoplastic myeloradiculitis associated with anti-GM2 antibodies. The woman presented with facial nerve palsy, positive pyramidal tract signs, and atrophic tetraparesis. Neuroimaging came back normal, but the spinal axis MRI was indicative for an inflammatory cause. The underlying primary tumor was a luminal B-like, non-specific type breast cancer stage IIB. Initially, she underwent neoadjuvant chemotherapy with doxorubicin and cyclophosphamide, followed by weekly paclitaxel. Then she was treated with a segmental resection and axillary dissection with adjuvant antihormonal therapy with a CDK4/6 inhibitor, followed by breast and lymphatic drainage radiation therapy with boost. From a neurological perspective, treatment of the myeloradiculitis with IVIG was administered prior to chemotherapy and then paused, to be taken up again during the adjuvant radiation therapy. The sensory-motor symptoms in the lower extremities were stable, with a slight increase in unilateral hand weakness. Unfortunately, the cancer recurred after 1 year and was treated palliatively with trastuzumab deruxtecan and another round of radiation therapy. Treatment course was complicated by a pulmonary embolism followed by a second local recurrence after 9 months, which was treated with sacituzumab govitecan and another course of radiation therapy. Due to the patient’s declining general condition, the therapy had to be discontinued and she passed away shortly thereafter.

## Literature search strategy

To identify relevant literature for this literature review, a structured search was conducted in the electronic databases PubMed and MEDLINE for publications in English or German up to January 16, 2026. The search terms used were “paraneoplastic syndrome” AND (“breast cancer” OR “mamma carcinoma” OR “mammary carcinoma” OR “mamma cancer” OR “mammary cancer” OR “gynecologic” OR “gynecology”). Inclusion criteria required original research (prospective or retrospective), reporting on patients with neurological PNS and underlying breast or other gynecological malignancies. Single-subject case reports were excluded. Such reports tend to describe atypical or extreme clinical presentations. By restricting the inclusion to case series (≥ 2 patients), we aimed to mitigate potentially misleading representation and focus on literature that facilitates the identification of clinical patterns and standardized diagnostic pathways, aligning with the primary objective of this review.

Data extraction included patient demographics, oncological characteristics (histology, stage), the temporal relationship between PNS and malignancy, clinical course, PNS type, radiologic findings and autoantibody profiles. Screening and extraction were performed by a primary reviewer, with ambiguous cases resolved through consensus with a second investigator. The primary reviewer screened titles and abstracts, and full-text articles were retrieved when abstract information was insufficient to determine eligibility. This was followed by the full-text review of all potentially eligible studies. Duplicates were flagged by the reference management tool and individually verified and removed. Due to the descriptive nature of the included data, results were synthesized by reporting the distributions of the outcomes of interest and illustrating them in a summary table.

## Clinical findings from the literature

**Fig. 3:**
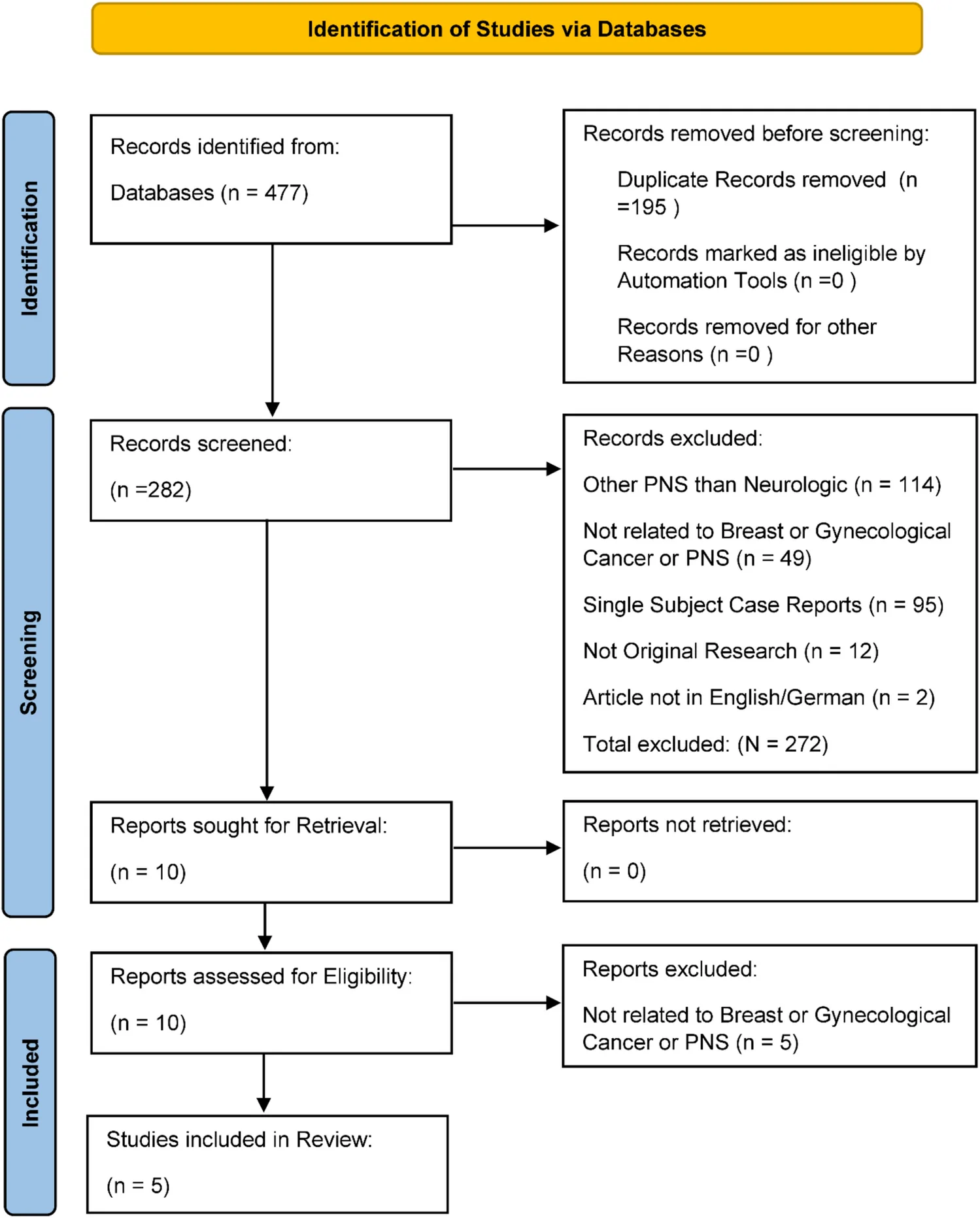
Flow diagram of the literature review

The initial search yielded 477 records. After removal of duplicates and screening against eligibility criteria, 5 studies comprising 72 patients were included (Fig. 3, Table 2). The studies were published between 1994 and 2025, with sample sizes ranging from 2 to 56 patients (median: 3 patients). All included studies employed a retrospective, descriptive design.

**Table 2:** Systematic literature review

Title	Authors and year of publication	Sample size^a^	Data collection period	Mean age [years]	Breast cancer [# cases]	Ovarian/primary peritoneal cancer [# cases]	Neurological PNS^a^	Antibodies	Timing of diagnosis
Paraneoplastic cochleovestibulopathy associated with breast cancer: a case report of two patients and literature review	Yang et al. 2025 [6]	2	Information not provided	47	2	0	Paraneoplastic cochleovestibulopathy (*n* = 2)	Negative in one and unknown in other case	1 st) PNS^b^, 2nd) cancer (*n* = 2)
Paraneoplastic syndromes associated with gynecologic neoplasms: experience from a tertiary care center in South India	Sudhakaran et al. 2024 [9]	2	2014—2021	53.4	0	2	Paraneoplastic cerebellar degeneration (*n* = 1), sensory neuropathy (*n* = 1)	anti-Yo antibodies (*n* = 1; paraneoplastic cerebellar degeneration), no information provided for sensory neuropathy	1 st) PNS^b^, 2nd) cancer (*n* = 1), paraneoplastic cerebellar degeneration), not reported for sensory neuropathy
Anti-Yo-associated paraneoplastic cerebellar degeneration: case series and review of literature	Chatham et al. 2021 [10]	3	Information not provided	65.66	2	1	Paraneoplastic cerebellar degeneration (*n* = 3)	Anti-Yo antibodies (*n* = 3)	1 st) PNS^b^, 2nd) cancer (*n* = 1), simultaneous (*n* = 2)
Breast cancer-related paraneoplastic neurologic disease	Murphy et al. 2018 [7]	56	1997—2016	52.8	56	0	Cerebellar ataxia (*n* = 14), myelopathy (*n* = 11), myopathy (*n* = 11), stiff-person syndrome (*n* = 10), non-chemotherapy-associated peripheral neuropathy (*n* = 8), brainstem encephalitis (*n* = 7), seizure (n = 4), myoclonus (*n* = 3), optic neuropathy (*n* = 3), encephalopathy (*n* = 3), neuropathic itching (*n* = 3), limbic encephalitis (*n* = 2), neuromuscular junction disorder (*n* = 2), polyradiculoneuropathy (*n* = 2), trigeminal neuropathy (*n* = 1), sensorineural hearing loss (*n* = 1), dystonia (*n* = 1), retinopathy (*n* = 1), radiculopathy (*n* = 1), anterior horn cell disorder (*n* = 1), parkinsonism (*n* = 1)	53.6% of patients with autoantibodies: anti-Yo (*n* = 10), anti-amphiphysin-IgG (*n* = 9), anti-Ri (*n* = 5), anti-AChR (binding) (*n* = 2), anti-AMPAR^c^ (*n* = 1), anti-N-type VGCC^d^ (*n* = 1), anti-GAD65^e^ (*n* = 1) and unclassified antibody (*n* = 1)	1 st) PNS^b^, 2nd) cancer (*n* = 32), 1 st) cancer, 2nd) PNS^b^ (*n* = 22), simultaneous (*n* = 2)
Paraneoplastic sensorimotor neuropathy associated with breast cancer	Peterson 1994 [8]	9	1982—1994	50.11	9	0	Paraneoplastic sensorimotor neuropathy (*n* = 9)	Information not provided	1 st) PNS^b^, 2nd) cancer (*n* = 6), 1 st) cancer, 2nd) PNS^b^ (*n* = 3)

*Sample size*^a^ refers to only the cases meeting the inclusion criteria, *PNS*^b^ paraneoplastic syndrome, *anti-AMPAR*^c^ anti-alpha-amino-3-hydroxy-5-methyl-4-isoxazolepropionic acid receptor, *anti-N-type VGCC*^d^ anti-N-type voltage-gated calcium channel, *anti-GAD65*^e^ anti-glutamic acid decarboxylase-65

The distribution of different cancer types in this structured review is largely influenced by the largest study, encompassing 56 patients with breast cancer and PNS. Therefore, breast cancer was the predominant underlying malignancy (95.83%, *n* = 69), while other gynecological cancers were a minority in the retrieved literature (4.17%, *n* = 3). The most frequent neurological manifestations were sensorimotor neuropathy (*n* = 28), followed by cerebellar ataxia (*n* = 18), myelopathy (*n* = 11), stiff-person syndrome (*n* = 10), and encephalitis (n = 7). Additional manifestations identified in the systematic review included paraneoplastic cochleovestibulopathy manifesting predominantly as sensorineural hearing loss. While Yang et al. [5] specifically described these manifestations, Murphy et al. [4] reported a broader spectrum of specific neurological deficits, including seizures and neuropathic itching. The clinical manifestations differed according to the diverse neurological PNS types. Patients suffering from paraneoplastic cerebellar degeneration presented with motor weakness, difficulty swallowing, lightheadedness, sudden progressive worsening in coordination, dysarthria, vertigo, nausea, diplopia, ataxia [4, 7, 8]. As for the paraneoplastic sensorimotor neuropathy, patients suffered from numbness and paresthesia on extremities, as well as on trunk and face, itching, muscle weakness as well as stiffness, painful muscle cramps and shooting pains [4, 9]. Paraneoplastic cochleovestibulopathy manifested itself as hearing loss, vertigo, unsteady gait, changed speech, and ataxia [5].

Specific antibodies were identified in 47.22% of patients. Anti-Yo was the most prevalent (42.4%), followed by anti-amphiphysin (27.3%) and anti-Ri (15.2%). Within the largest cohort, Murphy et al. [4] successfully identified autoantibodies in 53.6% of their 56 participants. In some instances, antibodies were not reported at all, as seen in Peterson et al. [9], or the reporting was incomplete, such as in Sudhakaran et al. [7], where results were provided for only one of two patients. Conversely, Chatham et al. [8] documented the antibody status for all three of their participants. Yang et al. [5] specifically noted that while an antibody search was conducted, a specific antibody could not be identified in one case despite immunofluorescence staining suggesting the presence of neuronal autoantibodies; in their second case, the search was entirely negative.

In the majority of cases (59.2%), neurological symptoms preceded the cancer diagnosis. Concurrent diagnoses occurred in 5.6%, and cancer was diagnosed first in 35.2%. Treatment outcomes (available for *n* = 66) were generally poor: no patient achieved complete recovery. While 16.67% showed a robust response and 40.91% exhibited mild to moderate improvement, 28.79% showed no clinical benefit, and 4.55% experienced progressive deterioration despite therapy.

Different from our case series, neurologic imaging, where reported, did not reveal any abnormalities. Murphy et al. [4] and Yang et al. [5] reported a diagnostic framework, with the other studies reporting which tests were done and sometimes explicitly mentioning which differential diagnoses were excluded. According to Murphy et al. [4], a paraneoplastic neurologic disease (PND) should be suspected in patients presenting with a subacute onset of neurological symptoms affecting the neuraxis without a clear explanation. This diagnostic workup must include autoantibody testing in both serum and CSF, ancillary testing for PND, a review of constitutional symptoms, and an assessment of the patient’s age-appropriate cancer screening status and consider a body CT or CT–PET imaging. An early referral to a neurologist familiar with PND to coordinate the evaluation and treatment is recommended. Yang et al. [5] present a framework to differentiate between paraneoplastic cochleovestibulopathy (PCVP) and primary autoimmune vestibulopathy. They differ by their etiology—remote malignant effects versus systemic or organ-specific autoimmunity—and the presence of neurological “red flags” in PCVP, such as cerebellar ataxia or dysarthria. While positive onconeural antibodies are characteristic of PNS, seronegative cases are diagnosed based on clinical features and a poor response to corticosteroids, which typically benefit primary autoimmune disorders. In female patients presenting with progressive cochleovestibular symptoms and ataxia, Yang et al. [5] recommend monitoring for underlying breast cancer.

Other mentioned differential diagnoses reported in the studies included were autoimmune demyelinating polyneuropathy, syphilis, HIV, vitamin B12 deficiency, and vascular affections for cerebellar degeneration [7, 8]. For PCVP, other diagnoses considered were Menière’s disease, direct neoplastic involvement of the nervous system, treatment-induced neurotoxicity, metabolic disturbances, infections as well as the beforehand mentioned primary autoimmune vestibulopathy [5]. For paraneoplastic sensorimotor neuropathy, specific testing was performed for thyroid function disorders, syphilis, vitamin B12 deficiency, Lyme disease, heavy metal exposure and compression of nerve roots or carpal tunnel syndrome [9].

## Discussion

The introductory case series and structured review provide an updated overview of the clinical presentation and management of neurological PNS in gynecological and breast cancer, highlighting their rarity and complexity.

In most cases—both published and in our own patients—the neurological syndrome preceded the diagnosis of the associated cancer, highlighting the need for early oncological workup in women with a suspected PNS. Across the literature and our own case series, the most commonly identified antibody was anti-Yo. Of the four PNS types presented in our case series, paraneoplastic cerebellar ataxia and encephalitis were also identified in the systematic review. The other two—acute myeloradiculitis and meningoencephalitis—were not represented in the included studies but are worth discussing.

### Anti-Yo paraneoplastic cerebellar degeneration

Anti-Yo paraneoplastic cerebellar ataxia, often referred to in the literature as paraneoplastic cerebellar degeneration (PCD), is linked to anti-Yo antibodies in about half of the cases [10]. In breast and ovarian cancer, cerebellar degeneration-related protein 2 (CDR2) is overexpressed, triggering an immune response that also targets the cerebellum, where CDR2 is normally expressed in Purkinje cells [11]. PCD in women is, therefore, primarily associated with ovarian and breast cancer [12, 13].

### Paraneoplastic encephalitis

In both our case series and in the literature, paraneoplastic encephalitis is frequently associated with anti-amphiphysin antibodies. Amphiphysin, a protein involved in clathrin-mediated endocytosis, is expressed in breast tumors and plays a role in inhibitory neurotransmission. Its autoimmune targeting can lead to various neurological manifestations, including limbic encephalitis and stiff-person syndrome [14, 15]. In the case presented, clinical improvement occurred with significant latency following the treatment of the underlying malignancy and after discontinuation of IVIG therapy. Anti-amphiphysin-associated neurological PNS are documented to be responsive to chemotherapy, corticosteroids, and immunotherapy, distinguishing them from many other, more treatment-refractory neurological PNS [16]. While clinical recovery can span months or years, the assessment of therapeutic success is complicated by documented instances of spontaneous improvement [16, 17].

### Acute myeloradiculitis

This condition is characterized by rapid-onset inflammation of the spinal cord and nerve roots, presenting with symptoms such as urinary retention, paresthesia, and muscle weakness [14, 18]. Although rare, it has been associated in isolated reports with a variety of cancers, including those of the lung, breast, kidney, thyroid, ovary, endometrium, and melanoma [19]. Antibody profiles remain inconsistent but include anti-amphiphysin, Collapsin response mediator protein 5 (CRMP5) [19, 20], glial fibrillary acidic protein (GFAP) [21], anti-Hu, anti-Yo, Purkinje cell cytoplasmic antibody type 2 (PCA2) and Kelch-like protein 11 (KLHL11) [20].

### Paraneoplastic meningoencephalitis

Meningoencephalitis is most commonly caused by infectious agents and only rarely has an autoimmune or paraneoplastic origin [22]. Accordingly, the literature is very limited. One case report described anti-amphiphysin-positive meningoencephalitis in a patient with PNS due to an underlying breast cancer [23]. We also identified a study reporting about 24 cases of meningoencephalitis in the context of checkpoint inhibitor therapy for cancer, which is not technically paraneoplastic. It remains unclear whether the two mentioned gynecologic cancer types contributed to the cases of meningoencephalitis in that cohort [24].

### Clinical implications and diagnostic considerations

PNS associated with gynecologic and breast cancers represent a highly diverse group of disorders. A wide range of symptoms, the rarity of their occurrence, and the frequent manifestation prior to tumor diagnosis often result in delayed diagnosis [10]. The timing of PNS diagnosis is crucial, as prompt treatment is essential for prognosis. Diagnostic workup can vary, depending on the suspected PNS type; however, a thorough search for antibodies is always recommended [14]. Aggressive tumor screening should include a CT scan of the abdomen and thorax, followed by a PET–CT if the results are negative. For suspected breast cancer, a mammography followed by an MRI is recommended. If no tumor is detected, imaging should be repeated every 3–6 months for up to 4 years [25]. For cases like paraneoplastic subacute cerebellar degeneration with anti-Yo antibodies and negative initial tumor search, an exploratory laparoscopy should be considered due to the high likelihood of an underlying ovarian carcinoma [26]. More specific testing might be necessary for exclusion of relevant differential diagnosis, depending on the clinical presentation. For paraneoplastic sensorimotor neuropathy, this might include imaging of the spine in search for nerve root compressions, or for PCVP assessing treatment response to corticosteroids, which might favor a primary autoimmune vestibular disorder. Graus et al. [6] introduced the Paraneoplastic-Neurologic-Syndrome-Care Score to standardize the diagnosis of paraneoplastic neurologic syndromes by categorizing clinical phenotypes and antibodies into high-risk and intermediate-risk levels. The system establishes three levels of diagnostic certainty—definite, probable, and possible—calculated from the clinical phenotype and antibody risk category and presence of cancer. Beforehand, alternative causes need to be excluded. The clinical course of PNS can vary substantially, with three of our four cases falling within the 28.79% of patients who show no symptom improvement and one case exemplifying the 40.91%, who exhibit mild to moderate improvement. The underlying biological mechanisms behind these syndromes remain poorly understood. It is still unclear whether the emergence of neoantigens on the tumor, which may trigger autoimmune responses, is driven by specific molecular events within the tumor. The variety of histological types associated with different paraneoplastic syndromes suggests that a single molecular mechanism is unlikely [10]. A multidisciplinary approach is essential for coordinating, for example, immunosuppressive therapy with surgical procedures or balancing oncologic treatment with neurological stabilization.

### Limitations

Several limitations must be acknowledged. The evidence base is constrained by small sample sizes and the retrospective design of included studies. Our findings rely heavily on one large cohort encompassing only breast cancer patients [4] and an older case series [9], although antibody identification was already established at that time [27].

These challenges further underscore the urgent need for large, international cohorts to research these rare conditions.

## Conclusion

Paraneoplastic syndromes in gynecologic and breast cancer remain underreported and insufficiently understood. Timely identification and multidisciplinary care are vital to improve patient outcomes. Diagnosis must trigger immediate screening for underlying malignancies. Further research is essential to optimize management, and pooling individual case reports in large international cohorts is crucial to improve the understanding of this rare condition.

## Data Availability

The datasets generated during and/or analyzed during the current study are available from the corresponding author on reasonable request.
